# Exploring Skull Base Osteomyelitis: Comprehensive Case Reports and Management Strategies

**DOI:** 10.7759/cureus.51973

**Published:** 2024-01-09

**Authors:** Nur Farahin Rosdi, Asma Abdullah, Thean Yean Kew, Juliana Fairuz Maktar, Farah Liana Lokman

**Affiliations:** 1 Otolaryngology - Head and Neck Surgery, Universiti Kebangsaan Malaysia Medical Centre, Kuala Lumpur, MYS; 2 Radiology, Universiti Kebangsaan Malaysia Medical Centre, Kuala Lumpur, MYS

**Keywords:** high-resolution computed tomography scan, facial nerve paralysis, hoarseness, otitis externa, skull base osteomyelitis

## Abstract

Skull base osteomyelitis is a not commonly encountered but potentially fatal consequence of untreated necrotizing otitis externa. Early recognition and appropriate treatment are crucial to prevent serious complications such as cranial nerve palsies, meningitis, and intracranial abscess formation. The case reports presented in this study provide a rich depiction of the clinical presentation, diagnostic challenges, and interventions employed. Early recognition and appropriate management of skull base osteomyelitis are crucial to prevent complications and improve patient outcomes.

## Introduction

Skull base osteomyelitis is an uncommon occurrence and serious infection involving the bones at the base of the skull. Although relatively uncommon, this condition requires prompt recognition and appropriate management to prevent neurological deficits, cranial nerve palsies, and potentially life-threatening complications.

Skull base osteomyelitis can have various etiologies, with the most common being a spread of infection from adjacent structures such as the paranasal sinuses, middle ear, or mastoid [1]. Other potential causes include direct trauma to the skull, postsurgical complications, and hematogenous spread from distant sources [1]. The condition is commonly observed in immunocompromised individuals [2]. They are particularly susceptible to developing skull base osteomyelitis. Additionally, facial nerve paralysis is common in this disease and is associated with its severity and mortality [2].

This report aims to provide a comprehensive exploration of skull base osteomyelitis through the presentation of two case reports and discuss the management strategies.

## Case presentation

Case one

A 64-year-old Malay male with poorly controlled diabetes, hypertension, and ischemic heart disease had been diagnosed with right necrotizing otitis externa and completed a three-month course of antibiotics. He presented with progressive difficulty in swallowing (dysphagia) and hoarseness.

**Figure 1:**
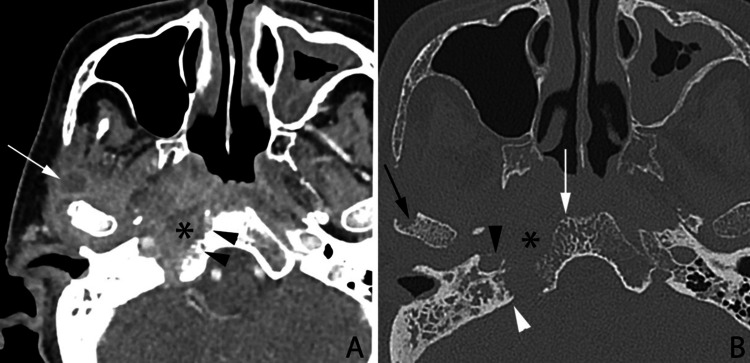
Axial contrast CT of the base of the skull in soft tissue (A) and bone algorithm (B) windows. Diffuse heterogeneously enhancing soft tissue with ill-defined margins is detected at the right temporomandibular joint, masticator, parapharyngeal, prevertebral spaces, and right nasopharynx, with suspicious encroachment upon the right jugular fossa. There is some suggestion of hypodensity within the right preclival region (asterisk in A), possibly due to early liquefaction. A discrete rim-enhancing ovoid lesion with fluid attenuation content centrally at the region of the right masseter indicates an abscess (white arrow in A). Superiorly, the extension of the said soft tissue enhancement is identified at the right foramen lacerum, with an erosion of the adjacent right carotid canal (black arrowhead in B), and cortical destruction at the right aspect of adjacent basisphenoid (black arrowheads in A). Bone algorithm CT displays lytic change within the right aspect of the basisphenoid marrow (white arrow in B), as well as erosion at the right jugular fossa (white arrowhead in B), occipital condyle, and hypoglossal canal (not shown). Note the eroded right petrous apex (asterisk in B, compared to the normal left counterpart). The rest of the right sigmoid sinus opacified unremarkably.

On clinical examination, he appeared pale and dehydrated. Examination of the ear revealed an edematous right external ear canal with a small tympanic membrane perforation. A flexible naso-pharyngeal-laryngoscope revealed paralysis of the right vocal cord, with the pooling of saliva in the pyriform sinus. High-resolution computed tomography (HRCT) of the temporal bone showed extensive osteomyelitis changes (Figure 1).

He was started on intravenous ciprofloxacin at a dosage of 400 mg per day. He completed a four-week course of intravenous ciprofloxacin but was switched to intravenous ceftazidime based on sensitivity test results. The change was prompted by sensitivity testing, which revealed resistance to ciprofloxacin. *Pseudomonas aeruginosa* was identified as the causative pathogen. He was also directed to collaborate with various medical specialties and allied health teams to optimize his overall well-being and address underlying health issues, including diabetes, end-stage renal disease, and diet. In addition, injection laryngoplasty was performed to medialize the position of paralyzed vocal cords, and swallowing rehabilitation was performed by a Speech-Language Therapist. He subsequently completed a total of 12 weeks of antibiotic treatment. Currently, he is in a stable condition, with resolved ear symptoms, experiencing improved clarity in voice and tolerating oral feeds.

Case two

A 70-year-old male presented with right ear discharge for a few months, which had been intermittent and accompanied by reduced hearing and headaches. The patient denied experiencing vertigo or facial asymmetry. He was a known poorly controlled diabetic with hypertension, ischemic heart disease, congestive heart failure, and chronic kidney disease. He underwent an uncomplicated combined approach tympanoplasty. Subsequently, he presented with a similar presentation over the left ear, which required a left cortical mastoidectomy. The surgical intervention focused on creating ventilation in the middle ear cleft, debriding soft tissue within the mastoid air cell, and addressing mastoiditis to facilitate the drainage of septic foci.

He developed complications in the form of a left cerebellar pedicle abscess with chronic skull base osteomyelitis. He completed six weeks of antibiotic treatment, which involved intravenous ceftriaxone for two weeks, followed by four weeks of oral cefuroxime and metronidazole. The histopathology report of the intraoperative specimen showed features of inflammation. An initial culture of the otorrhea showed no isolated pathogens.

He had been well for a few months. However, he presented again with an episode of neck pain, and upon further examination, he was diagnosed with skull base osteomyelitis with cervical vertebra involvement. Otoscopy findings did not show evidence of infection. Examination of the cranial nerves was unremarkable. The neck range of movement was limited due to pain. A computed tomography (CT) scan showed progressive inflammatory changes.

**Figure 2:**
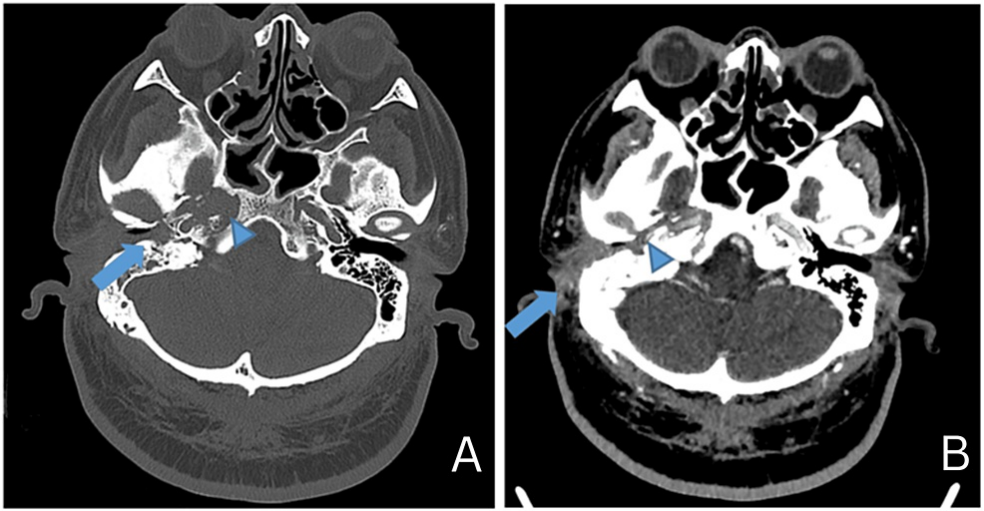
(a) Axial contrast-enhanced CT image in the bone window shows erosions at the floor of the osseous right external auditory canal (arrow) with erosions at the condylar head of the right mandible and mandibular fossa (arrowhead). (b) Axial contrast-enhanced CT image in the soft tissue window shows smooth diffuse mucosal thickening and enhancement of the cartilaginous and osseous segments of the right external auditory canal (arrow) with enhancing soft tissue mass within the right temporomandibular joint (arrowhead).

**Figure 3:**
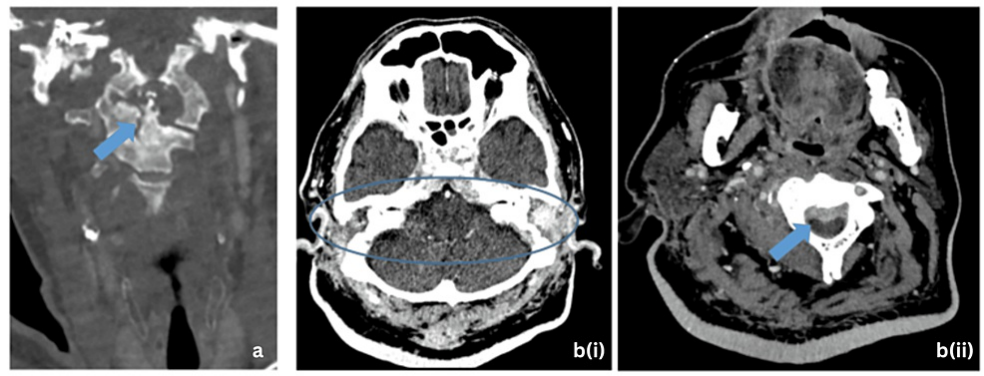
(a) Coronal contrast-enhanced CT image in the bone window shows further progression of the cortical erosion of the basisphenoid, clivus, dens of C2 vertebra (arrow), and the posterior aspect of C3 and C4 vertebra bodies. (b)(i) Axial contrast-enhanced CT image in soft tissue window shows smooth diffuse mucosal thickening and enhancement of the cartilaginous and osseous segments of the bilateral external auditory canal (circle) (b)(ii) with prevertebral soft tissue enhancement causing mild spinal canal narrowing anteriorly, worst at the C2 vertebral level (arrow). These findings were interpreted as progressive inflammatory changes.

Additionally, it was complicated by spinal canal stenosis at the C2 level and dehiscence of the left tegmen tympani, indicating worsening osteomyelitis of the skull base. HRCT of the temporal bone showed extensive disease involvement (Figures 2, 3).

He completed three weeks of antibiotic treatment, which consisted of one week of oral ceftriaxone, followed by two weeks of oral cefuroxime. After completing the treatment, he was discharged home in a stable condition, being asymptomatic with no ear discharge and showing no signs of infection. He continued with regular follow-ups.

## Discussion

Skull base osteomyelitis is a rare and severe condition characterized by inflammation and infection of the bones forming the base of the skull. In the above cases, skull base osteomyelitis developed as a complication of chronic otitis media and mastoiditis. The involvement of the skull base presents a significant challenge due to the proximity to critical structures and the potential for serious complications, including brain abscesses and cranial nerve palsies.

Skull base osteomyelitis typically develops gradually, initially presenting as a prolonged case of otitis externa that persists for several weeks or even months. Infections originating from the external ear can extend into the surrounding temporal bone through the fissures of the Santorini external auditory canal (EAC) and the osseocartilaginous junction of the EAC. From there, the infection can further spread through the compact bone’s Haversian system, affecting the entire skull base and cervical vertebrae [1]. The occurrence of infection extending to the cervical spine in individuals diagnosed with skull base osteomyelitis is uncommon, with only a limited number of such cases documented in the existing literature [3].

The presence of cranial nerve palsy in patients with otitis externa raises suspicion of an underlying skull base osteomyelitis [4]. The presence of vocal cord palsy indicates involvement of the cranial nerves, further highlighting the severity and other potential complications of skull base osteomyelitis. Early identification of cranial nerve involvement is important for appropriate management and to prevent long-term sequelae.

A comprehensive evaluation, including imaging studies and further diagnostic procedures, is crucial to determine the extent of the infection and associated complications. Radiologic evaluation plays a critical role in the diagnosis of skull base osteomyelitis. CT best demonstrates cortical and trabecular destruction of the bone. HRCT offers the capability of reformatting images in multiple planes. It is considered the preferred imaging modality for the identification of cortical bone erosion or trabecular demineralization associated with osteomyelitis. When dealing with suspected skull base osteomyelitis, it is crucial to evaluate cortical bone loss, which can sometimes be subtle. Various critical areas should be thoroughly examined, including the bony EAC, mastoid tip, temporomandibular joint, petrous apex, petro-occipital fissure, foramen lacerum, jugular foramen, and clivus [5].

Treatment of skull base osteomyelitis typically involves a combination of surgical intervention and prolonged antibiotic therapy. Surgical options may include debridement of infected tissue, mastoidectomy, and skull base reconstruction if necessary. Surgical debridement can effectively eliminate non-viable tissue and decrease the infection load, facilitating better penetration of antibiotics.

Typically, patients start with intravenous therapy and subsequently transition to long-term oral medication, ensuring an appropriate clinical response. Due to the increased susceptibility to polymicrobial infections, it is advisable to initiate multiple broad-spectrum antimicrobial therapies promptly, pending the availability of microbiology and histopathology studies [1]. In recent decades, numerous studies have identified *Pseudomonas aeruginosa* infection as a prominent cause of skull base osteomyelitis, particularly in cases of necrotizing otitis externa [1,6]. Antibiotics should be tailored based on culture and sensitivity results to target the causative organisms. In a culture-negative result, treatment with broad-spectrum antimicrobials should be initiated with close evaluation of clinical response.

Based on previous studies, in the majority of patients, a minimum antimicrobial therapy duration of 6-12 weeks is necessary. Considering the complexity of skull base osteomyelitis, it is probable that treatment duration will need to be customized on a case-by-case basis. Close monitoring of the patient’s response to treatment is essential to ensure adequate control of the infection, resolution of symptoms, and prevention of complications.

## Conclusions

The cases presented in this study highlight the challenges and potential complications associated with skull base osteomyelitis. A comprehensive approach, including surgical intervention and targeted antibiotic therapy, is necessary to control the infection, minimize long-term sequelae, and prevent morbidity and mortality. Long-term follow-up is necessary to assess for any recurrence or residual deficits, such as cranial nerve dysfunction and progression of the disease.

## References

[1] Khan MA, Quadri SA, Kazmi AS (2018). A comprehensive review of skull base osteomyelitis: diagnostic and therapeutic challenges among various presentations. Asian J Neurosurg.

[2] Rothholtz VS, Lee AD, Shamloo B, Bazargan M, Pan D, Djalilian HR (2008). Skull base osteomyelitis: the effect of comorbid disease on hospitalization. Laryngoscope.

[3] Bruschini L, Berrettini S, Christina C (2019). Extensive skull base osteomyelitis secondary to malignant otitis externa. J Int Adv Otol.

[4] Mejzlik J, Cerny M, Zeinerova L (2019). The routes of infection spread in central skull-base osteomyelitis and the diagnostic role of CT and MRI scans. BMC Med Imaging.

[5] Chapman PR, Choudhary G, Singhal A (2021). Skull base osteomyelitis: a comprehensive imaging review. AJNR Am J Neuroradiol.

[6] Singh A, Al Khabori M, Hyder MJ (2005). Skull base osteomyelitis: diagnostic and therapeutic challenges in atypical presentation. Otolaryngol Head Neck Surg.

